# Cascade Deep Forest With Heterogeneous Similarity Measures for Drug–Target Interaction Prediction

**DOI:** 10.3389/fgene.2021.702259

**Published:** 2021-08-24

**Authors:** Ying Zheng, Zheng Wu

**Affiliations:** School of Computer & Communication Engineering, Changsha University of Science & Technology, Changsha, China

**Keywords:** drug repositioning, drug discovery, drug–target interaction, heterogeneous similarity measures, cascade deep forest

## Abstract

Drug repositioning is a method of systematically identifying potential molecular targets that known drugs may act on. Compared with traditional methods, drug repositioning has been extensively studied due to the development of multi-omics technology and system biology methods. Because of its biological network properties, it is possible to apply machine learning related algorithms for prediction. Based on various heterogeneous network model, this paper proposes a method named THNCDF for predicting drug–target interactions. Various heterogeneous networks are integrated to build a tripartite network, and similarity calculation methods are used to obtain similarity matrix. Then, the cascade deep forest method is used to make prediction. Results indicate that THNCDF outperforms the previously reported methods based on the 10-fold cross-validation on the benchmark data sets proposed by Y. Yamanishi. The area under Precision Recall curve (AUPR) value on the Enzyme, GPCR, Ion Channel, and Nuclear Receptor data sets is 0.988, 0.980, 0.938, and 0.906 separately. The experimental results well illustrate the feasibility of this method.

## Introduction

In the past few decades, investment in drug research and development has grown rapidly, but most drugs have failed in the first phase of clinical trials. Moreover, it normally costs billions of dollars and consumes 10 years for any drug to be put on the market completely (Roessler et al., 2021). At present, drug repositioning has a wide prospect and provides evidence for further drug discovery, whose purpose is to determine potential therapeutic targets for existing drugs, thereby saving time and minimizing risks of conventional drug development (Stein et al., 2021).

The key of drug repositioning hinges on identifying drug–target interaction (DTI), which exerts a vital role in drug research and development (Badkas et al., 2020). Currently, traditional experimental approaches are either time consuming or high costly. Despite that potential drug indications can be directly detected by target or cell screening of thousands of drugs in synthetic databases, there are still hurdles to massively relocate drugs owing to the needs of collecting existing drugs, specialized equipment, and screening tests (Turanli et al., 2018).

In general, the traditional methods for calculating drug target interactions mainly consist of ligand method and structure method (Huang et al., 2020; Yang et al., 2020; Zhang et al., 2020). The ligand-based methods predict potential DTI *via* contrasting candidate ligands with known ligands capable of binding to them, but it does not perform well in the absence of ligand information for potential targets (Juárez-Saldivar et al., 2020). The structure-based method mainly uses the docking simulation technology to predict the potential DTI on the basis of known three-dimensional structure. In the same way, this method that relies on simulated docking’s reliability often consumes a plenty of time and requires all drugs and targets to provide accurate and reliable three-dimensional structure (Vivarelli et al., 2020).

Along with sustainable innovative developments of biological data, and high-speed improvements of machine learning technology in recent years, a variety of methods for computational drug repositioning have been put forward correspondingly and achieved some achievements in practical applications (Lan et al., 2016, 2020; Chen et al., 2021; Li et al., 2019; Liu et al., 2019; Zeng et al., 2019; Fahimian et al., 2020; Rauschenbach et al., 2020; Zhou et al., 2020; Jarada et al., 2021; Meng et al., 2021). Machine learning is a beneficial complement to ligand-based and structure-based methods. It has been widely developed and applied as an effective method for pinpointing drug–targets as well as predicting drug-diseases. Machine learning is able to systematically integrate biological databases, with the purpose of predicting potential DTI and drug–disease interactions.

The method of similarity constrained probabilistic matrix factorization (SCPMF) is used for drug repositioning through recognizing novel drug–virus coactions (Meng et al., 2021). Moreover, SCPMF innovatively reconstructs the drug–virus interaction matrix, by dexterously projecting the drug–virus interaction matrix into two potential feature matrices for viruses and drugs. A new framework named Similarity Network Fusion and Neural Networks (SNF-NN) on the basis of deep learning was proposed and elaborated, which predicts new drug–disease interactions though using similarity selection relevant to drugs and diseases, similarity network fusion, and a novel neural network model with superior tuning (Jarada et al., 2021). By comparison of the performance of SNF-NN with that of nine benchmark machine learning methods, the robustness of SNF-NN is calculated. The values of AUC and AUPR are 0.867 and 0.876, respectively. Besides, a previous study has shown that a method based on network called RepCOOL is utilized for drug repositioning (Fahimian et al., 2020). The eventual model of drug repositioning is constructed on account of a random forest classifier. RepCOOL recommends four novel drugs for the treatment of breast cancer at stage II, namely, paclitaxel, doxorubicin, tamoxifen, and trastuzumab. In addition, a network embedding based method for predicting drug–disease interactions (NEDD) is raised (Zhou et al., 2020). Initially, through constructing a heterogeneous network and utilizing meta-paths of various lengths, NEDD accurately obtains the indirect associations between drugs and diseases or their strong proximity, thereby acquiring representation vectors of drugs and diseases with low dimensions. NEDD estimates novel relationships between diseases and drugs by utilizing a random forest classifier. A recent study has reported that a network-based method about deep learning for drug repositioning (deepDR) recognizes advanced characteristics of drugs from heterogeneous networks through a multi-mode autoencoder. Then, through a variational autoencoder, the obtained low-dimensional representation of the drug as well as clinically reported drug–disease pairs are uniformly encoded and decoded to infer candidates for approved drugs that were actually without initial approval (Zeng et al., 2019).

The main contributions of this paper are summarized as follows:

We study various calculation methods based on the tripartite heterogeneous network, and finally adopt the Gaussian kernel between each layer, and the Tanimoto’s coefficient is used in the drug layer to calculate the chemical structure similarity matrix. Besides, the similarity matrix is fitted by all matrices;

We improve and adjust the parameters according to the gcForest (Zhou and Feng, 2019) method. We use 10-fold cross-validation to check the final prediction (termed THNCDF, Tripartite Heterogeneous Network Cascade Deep Forest).

We compare the results of THNCDF with four types of methods (Cao et al., 2014; Hao et al., 2016; Rayhan et al., 2017; Thafar et al., 2020). The experimental results show that the THNCDF method has good performance, and the area under Precision Recall curve (AUPR) values on the four benchmark data sets reach 0.988, 0.980, 0.938, and 0.906.

The rest of this paper is organized as follows. In Section 2, we introduce the data sets used for similarity measurement, and then we present the general framework and cascade deep forest methods with details in Section 3. In Section 4, the performance of our proposed THNCDF method is evaluated through extensive experiments. At the end, some discussions are provided in Section 5.

## Related Work

### Data Sets

In our experiments, we use the data sets listed in Table 1 to build a tripartite heterogeneous network model. Table 1 shows the exactly biologic data sets we used during the experiments (Yamanishi et al., 2008; Zheng and Wu, 2021). Especially, the main resource of the data set for the disease layer is from DisGeNET. This paper also uses a data set called DisGeNET approved, which contains FDA-approved drugs and their corresponding protein targets in the DisGeNET.

**TABLE 1:** Sources and verification of databases.

Resource	Description	Url
DrugBank	Free accessible drug database	www.drugbank.ca/
DisGeNET	Free accessible human disease database	www.disgenet.org/
ChEMBL	Free accessible drug and target database	www.ebi.ac.uk/chembl/
Kegg	Free accessible database for molecular-level information	www.kegg.jp/
Uniprot	Free accessible protein sequence and annotation database	www.uniprot.org
OMIM	Free accessible compendium for Mendelian disorder	www.omim.org/

We will evaluate the performance of THNCDF on benchmark data sets. The benchmark data sets used in many DTI predictions were originally proposed by Y. Yamanishi, which have been considered as the golden data sets for comparing various DTI prediction methods. The benchmark data sets are listed in Table 2, which are downloaded from http://web.kuicr.kyotou.ac.jp/supp/yoshi/drugtarget/. The data sets include four subsets grouped by target classification: Enzyme, ion channel, GPCR (G protein-coupled receptor), and nuclear receptor. The largest subset, Enzyme, includes 445 drugs and 664 targets with 2,926 known DTI between them. Another NR, the smallest subset includes only 54 drugs and 26 targets with 90 known interactions. The other two subsets, IC and GPCR, consist of 210 and 223 drugs, 204 and 95 targets, and 1,476 and 635 known interactions, respectively.

**TABLE 2:** Benchmark data sets.

Data sets	Drugs	Targets	n_*d*_/n_*t*_	Interactions
Enzyme	445	664	0.667	2,926
Ion channel	210	204	1.03	1,476
GPCR	223	95	2.35	635
Nuclear receptor	54	26	2.08	90

### Tripartite Heterogeneous Network

Based on the related ideas of pharmacology, the therapeutic effect of a single drug is relatively limited for diseases that are complex multiple pathological (Zamami et al., 2017; Zhu et al., 2020). Recently, the development of high-throughput biotechnology has produced a large amount of data. However, one of the main difficulties is how to collect and analyze the required biomedical data because they are heterogeneous and the data generated from different experiments include different types of information, such as nucleotide sequences and protein–protein interactions (Luo et al., 2020).

**FIGURE 1:**
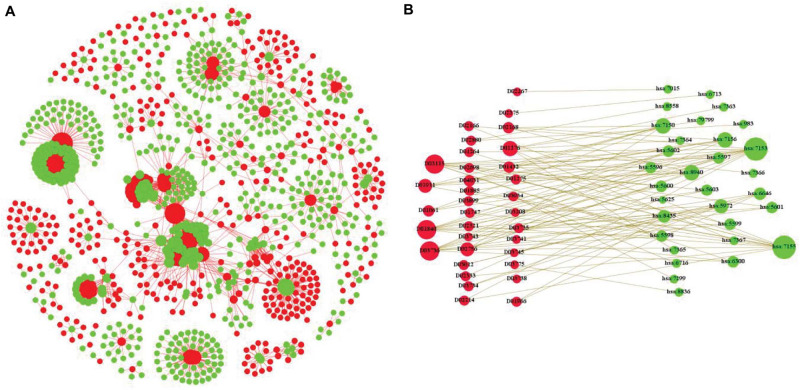
An example of bipartite graph for drug–target interactions. **(A)** Is the part of visualization of the Enzyme in benchmark data sets, in which the red nodes are drugs and the green nodes are targets. **(B)** Is the bipartite graph model of a part of Figure 1A.

In this paper, we integrate the composition of many different heterogeneous networks and construct our novel tripartite heterogeneous network model according to different types of data. Figure 1A is the part of visualization of the Enzyme in benchmark data sets, in which the red nodes are drugs and the green nodes are targets. Figure 1B is the bipartite graph model of a part of Figure 1A; the red nodes are drugs, and the green nodes are targets in the same.

We construct a tripartite network that includes three layers: drugs, targets, and diseases. Correspondingly, two types of interactions, drug–target interactions and target–disease interactions, are interpreted as edges to connect nodes in these layers. We mainly focus on constructing the similarity matrix and feature information of the tripartite heterogeneous network.

## Materials and Methods

In this study, we propose THNCDF, a new computational approach for molecular target identification from known drug–target centered DTI prediction. It utilizes low-dimensional but informative matrix representations of features for both drugs and targets through a cascade deep forest classifier in prediction of DTI (Zheng and Wu, 2021).

**FIGURE 2:**
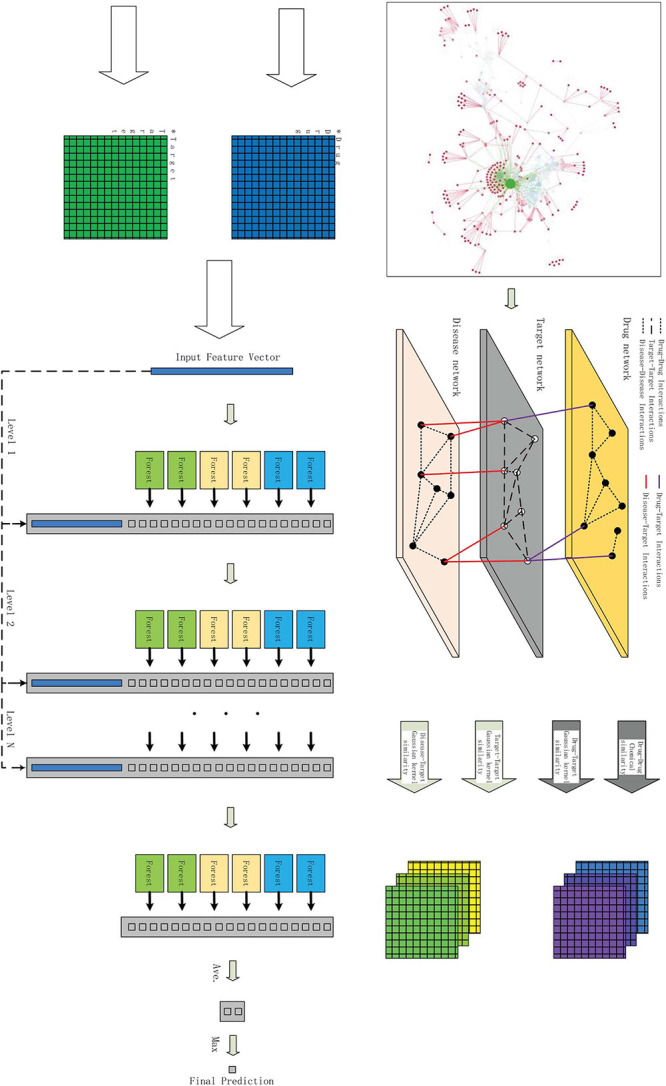
The overview of the proposed work THNCDF.

As shown in Figure 2, THNCDF mainly includes three steps: (1) Data integration and complete heterogeneous network is obtained, which contains diverse cheminformatics and bioinformatics profiles; (2) Similarity matrix calculation and parameter setting; (3) Application of cascade deep forest classifier and verification of the results.

### Similarity of Medicinal Chemical Structures

To ensure that the features in the network model are distinguishable, the similarity of the medicinal chemical structure is a relatively objective feature (Zheng and Wu, 2021). In particular, the chemical structure of various drugs under the same standard can be obtained through the simplified molecular-input line-entry system (SIMILES), and then converted into 166-bits string of a certain length fingerprint. Thus, each fingerprint represents a unique drug. Through the calculation of Tanimoto’s coefficient, the similarity matrix of medicinal chemical structure among all drugs is obtained. The formula for calculating Tanimoto’s coefficient is shown in Equation (1).

(1) $$S\,I\,M_{c\,h\,e\,m}=\frac{\left|f\,\left(d\,x\right)\times f\,\left(d\,y\right)\right|}{\left|f\,\left(d\,x\right)+f\,\left(d\,y\right)\right|-\left|f\,\left(d\,x\right)\times f\,\left(d\,y\right)\right|}$$

where *f*_(dx)_ is the binary chemical fingerprint of drug *x*. According to Equation (1), a matrix of chemical structure similarity is constructed.

### Gaussian Kernel Similarity

The Gaussian kernel is defined as the unimodal of the Euclidean distance between any two points in the network (Zheng and Wu, 2021). In THNCDF method, the Gaussian kernel is mainly used to calculate the feature of the connection between two layers, like the edge between the drug layer and the target layer or between the target layer and the disease layer. Also, for drug–drug interactions and target–target interactions, the Gaussian Kernel can calculate the edges in the same layer. Therefore, the calculation formula is commonly used to construct various types of matrices, such as the drug–drug interactions similarity matrix, target–target interactions similarity matrix, and target–disease interactions similarity matrix. The calculation formula is as follows:

(2) $$K_{G\,I\,P,d}\,\left(D_{i},D_{j}\right)=e\,x\,p\,\left(-\gamma_{d}\,{||y\,d_{i}-y\,d_{j}||}^{2}\right)$$

(3) $$\gamma_{d}=\gamma_{d}^{\prime }/\left(\frac{1}{m}\,\sum_{i=1}^{m}{||y\,d_{i}||}^{2}\right)$$

where *D_i_* is defined as the *i-th* drug in the drug set, *T_i_* represents the *i-th* target in the target set, while *ts*_*i*_ represents the *i-th* target in the target–disease interactions set. *m* is the size of drug set, while *n* and *k* represent the size of target set and the size of target–disease interactions set, respectively. The adjacency matrix *Y* ∈ *mn* represents the known drug–target interactions. If the drug and the target have an existing interaction, the value is 1; otherwise, the value is 0. *yd*_*i*_{*y*_*i*1_,*y*_*i*2_,…,*y*_*in*_} is defined as the correlation vector between the drug *d_i_* and all targets; meanwhile, *yts*_*i*_{*y*_*i*1_,*y*_*i*2_,…,*y*_*in*_} is defined as the correlation vector between the target *ts*_*i*_ and all diseases. γ_*d*_, γ_*t*_, and γ_*ts*_ are adjustment parameters that control the width of the kernel, where$\gamma_{d}^{\prime }$, $\gamma_{t}^{\prime }$, and $\gamma_{t\,s}^{\prime }$ are set to 1 by using Gaussian kernels.

### Similarity Matrix Fusion

According to the above multiple similarity matrices, we construct a kernel containing the spatial information of drugs and targets (Ding et al., 2018, 2020a,b; Zheng and Wu, 2021). Since the similarity matrix is not a positive definite matrix, predictions are ultimately required. We linearly fit the similarity matrix of drug chemical structure, the drug Gaussian kernel, the target Gaussian kernel, and the disease Gaussian kernel. We also set the weighted factors in the following equations empirically.

(4) $$S\,I\,M_{d\,r\,u\,g}\,\left(d_{x},d_{y}\right)=\left(1-\alpha \right)\times K_{G\,I\,P,d}\,\left(d_{x},d_{y}\right)+\alpha \times S\,I\,M_{c\,h\,e\,m}\,\left(d_{x},d_{y}\right)$$

(5) $$S\,I\,M_{t\,a\,r}\,\left(t_{x},t_{y}\right)=\left(1-\alpha \right)\times K_{G\,I\,P,t}\,\left(t_{x},t_{y}\right)+\alpha \times K_{G\,I\,P,S}\,\left(t\,s_{x},t\,s_{y}\right)$$

The result of similarity matrices is used as the original input of the next step. In the latter experiments, in order to balance the constructed similarity matrix, the ratio of 0.5:0.5 is used with parameter setting.

### Cascade Deep Forest

Random forest, developed by Bermain and Culter (Breiman, 2001), is widely used due to its excellent stability and resistance to overfitting. Nowadays, random forest has been successfully applied to the analysis of multiple biological and pharmacological contexts, such as Diabetic Retinopathy screening procedure (Alabdulwahhab et al., 2021) and detection of copy number variations for uncovering genetic factors (Zhuang et al., 2020). But in novel review by Zhou et al., deep learning based on non-differentiable modules exhibits the possibility of constructing deep models without using backpropagation. They have proposed the gcForest approach, which has generated three characteristics: layer-by-layer processing, in-model feature transformation, and sufficient model complexity. It provides an alternative methods to deep neural networks (DNNs) to learn hyper-level representations at a low computational cost. gcForest is a novel decision tree ensemble, with a cascade structure. It has much fewer hyper-parameters than DNNs, which the training process does not rely on backpropagation. In fact, the most important value of gcForest approach is it may open a door for non-NN style deep learning, or deep models based on non-differentiable modules. An extended depiction and the study of the theory on random forest or gcForest can be referred to the Web site of Bremain or the paper of Zhou et al.

Based on the advantage of random forest and characteristics of gcForest, we construct the THNCDF method, which includes the similarity matrices described above and utilizes improved gcForest approach for prediction. First, the fusion similarity matrix is the origin input for cascade structure of deep forest. Each level of cascade receives the feature information processed by its previous level and outputs its processing result to the next level. All level is an ensemble of decision tree forest. For example, each forest will count the percentages of different classes of training examples at the leaf node, and then average all trees in the same forest to obtain an estimate of the class distribution.

Secondly, we use three random forests: (a) two completely random tree forests, (b) two gradient boosting tree forests, and (c) two extra randomized tree forests. Each forest contains 1,000 trees, and there are 6,000 trees in total. Each node selects a feature randomly as the judgment condition and generates leaf nodes according to the condition. Stop until each leaf node contains only instances of the same class.

To compare with other results, we use 10-fold cross validation (Liu et al., 2016). It means that class vectors produced by each forest are generated by 10-fold cross validation to reduce the risk of overfitting. Finally, if there is no significant performance gain, the training process will terminate. The number of cascade levels is automatically determined.

## Experimental Results and Analysis

### Baseline Methods

In order to evaluate the performance of our method, we mainly introduce DTI prediction results compared with baseline methods on the benchmark data sets that are proposed by Y. Yamanishi. The following are the state-of-the-art methods made in comparison with the same standard criteria:

RLS-KF (Hao et al., 2016): A regularized least squares combining with nonlinear kernel fusion method is developed.

RF (Cao et al., 2014): A computational method integrated the information from network, chemical, and biological properties. This method is developed based on the random forest combining with integrated features.

DTiGEMS (Thafar et al., 2020): A computational method using graph embedding, graph mining, and similarity properties techniques. DTiGEMS firstly applies a similarity selection procedure and a similarity fusion algorithm. Then, it integrates multiple drug–drug similarities and target–target similarities into the final heterogeneous graph structure after.

iDTI-ESBoost (Rayhan et al., 2017): A prediction model uses evolutionary and structural features. The method uses a new data balancing and boosting technique to make prediction.

### Evaluation Criteria

Two quality measures are commonly used to evaluate the performance of these methods: AUC and AUPR. Specifically, we calculate the receiver operating characteristic curve (ROC) of true positive as a function of false positive, and use the area under the ROC curve (AUC) value as a quality measure. In addition, we also calculate the precision–recall curve (P–R), which is the chart of true positive rate between all positive predictions of each given recall rate. The area under the P–R curve (AUPR) provides a quantitative assessment. These two kinds of quality measures have become the standard criteria for evaluating methods.

### Prediction Ability

To provide a fair comparison of DTI prediction performances, we apply these methods on the same benchmark data sets. We also use 10-fold cross-validation random setting, the same evaluation criteria, and optimal parameters of each method.

**FIGURE 3:**
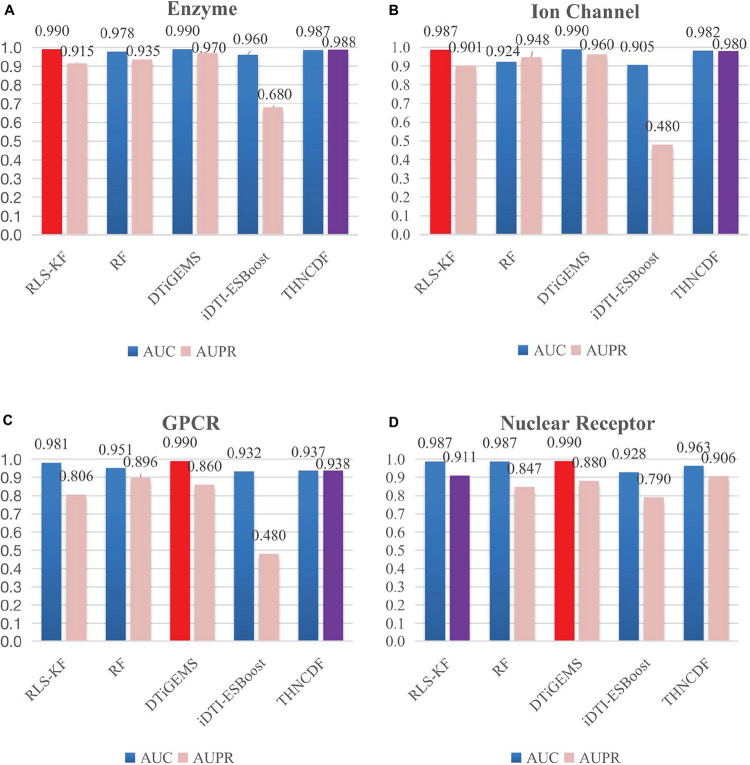
Comparison results for THNCDF and other methods in terms of AUC and AUPR values on the benchmark data sets. The best AUC values are indicated in red, and the best AUPR values are in purple.

From the results reported in Table 3 and Figure 3, THNCDF algorithm still maintains a high performance, especially for the AUPR values. For example, in the enzyme data set (Figure 3A), the ion channel data set (Figure 3B), and the GPCR data set (Figure 3C), THNCDF outperforms all other methods by achieving the best performance for AUPR values. On the other hand, for the AUC values, THNCDF still maintains the high performance. It is well known that the training of DNN usually requires a large amount of training data; hence, its implementation on tasks with small-scale data is not suitable. This is the inherently unavoidable characteristic of the method we use. Thus, it is reflected in the correlation between the size of the benchmark data sets and the AUC values obtained.

**TABLE 3:** The results of the baseline methods and the THNCDF method.

Data sets	Methods	RLS-KF	RF	DTiGEMS	iDTI-ESBoost	THNCDF
Enzyme	AUC	0.990*	0.978	0.990	0.960	0.987
	AUPR	0.915	0.935	0.970	0.680	0.988*
Ion channel	AUC	0.987	0.924	0.990*	0.905	0.982
	AUPR	0.901	0.948	0.960	0.480	0.980*
GPCR	AUC	0.981	0.951	0.990*	0.932	0.937
	AUPR	0.806	0.896	0.860	0.480	0.938*
Nuclear receptor	AUC	0.987	0.987	0.990*	0.928	0.963
	AUPR	0.911*	0.847	0.880	0.790	0.906

*For each methods, ^∗^ indicates the highest value.*

In addition, the number of positive samples and negative samples in each data set is highly imbalanced. The fact that few positive samples make THNCDF cannot exert its advantages, which is based on a large amount of training data. For benchmark data set, the feature dimension used in this method is low, and cascade deep forest has great advantages in the representation learning of ultra-high-dimensional data.

As shown in Figure 3, it is found that the prediction accuracy is approximately equal to each other. It also shows that the THNCDF preserves the best performance on all data sets so that it can be migrated to other predictions. The experiment procedure shows that THNCDF is not very sensitive to parameter settings. Therefore, it does not need large-scale parameter adjustment, especially the selection of the optimal combination of base classifiers. Comparing with DNN, THNCDF is more stable and easier.

It is worth mentioning that for the two commonly used evaluation metrics, more and more authors think that AUPR provides more informative assessment than AUC for highly imbalanced data sets. They argue in favor of AUPR values as a key standard of evaluating the performance for skewed data sets, especially the data sets with more negative samples than positive samples. In fact, all of the four subsets in the benchmark data sets possess the imbalanced characteristic, which means that the number of known drug–target interactions is far less than the number of pairs with no interaction evidence. So a more sensitive AUPR metric is generally preferred for assessing the prediction results for those imbalanced datasets. From this perspective, the result clearly shows that THNCDF outperforms the prediction in terms of AUPR as well.

## Discussion

In this paper, we present a new multi-kernel computational approach combined with an improved cascade deep forest, which leads to good predictive performance on the task of predicting DTI. The values of AUPR on four benchmark data sets are improved to 0.988, 0.980, 0.938, and 0.906, respectively. Theoretically, THNCDF can process various high dimensional features by utilizing heterogeneous networks. However, we still have some problems to be solved in the future. First, even though studies have discussed multiple similarity calculation methods, they have not escaped the research scope on the network interactions. We are more looking forward to the introduction of new biochemical similarity calculation methods or data sets. Secondly, we suggest applying different embedding techniques, integrating more similarity measures from more sources, and generating more graph-based features. It can also be found that various data sets, such as chemical structure, side effect, therapeutic effect, gene expression, drug binding site, and semantic data, have been utilized in former studies. However, the disadvantages of these biomedical data sets are also obvious, which include high data noise, incompleteness, and inaccuracy. Thirdly, some potential extensions of our work include applying THNCDF to different networks formulated as an interaction prediction problem. Popular examples of interaction prediction in the bioinformatics field include but are not limited to drug–drug interactions prediction, drug–disease interactions prediction, and gene–disease association prediction.

## Data Availability Statement

The original contributions presented in the study are included in the article/supplementary material, further inquiries can be directed to the corresponding author/s.

## Author Contributions

YZ and ZW: conceptualization and validation. YZ: methodology, writing—review and editing, and supervision. ZW: software and writing—original draft preparation. Both authors have read and agreed to the published version of the manuscript.

## Conflict of Interest

The authors declare that the research was conducted in the absence of any commercial or financial relationships that could be construed as a potential conflict of interest.

## Publisher’s Note

All claims expressed in this article are solely those of the authors and do not necessarily represent those of their affiliated organizations, or those of the publisher, the editors and the reviewers. Any product that may be evaluated in this article, or claim that may be made by its manufacturer, is not guaranteed or endorsed by the publisher.
